# Supplementary material for: An obligate symbiont of Haematomyzus elephantis with a strongly reduced genome resembles symbiotic bacteria in sucking lice
Source: Appl Environ Microbiol. 2025 May 14;91(6):e00220-25. doi: 10.1128/aem.00220-25 (PMC12175528; doi:10.1128/aem.00220-25)
Supplement: Figure S1 — Maximum likelihood tree. [file aem.00220-25-s0001.pdf]

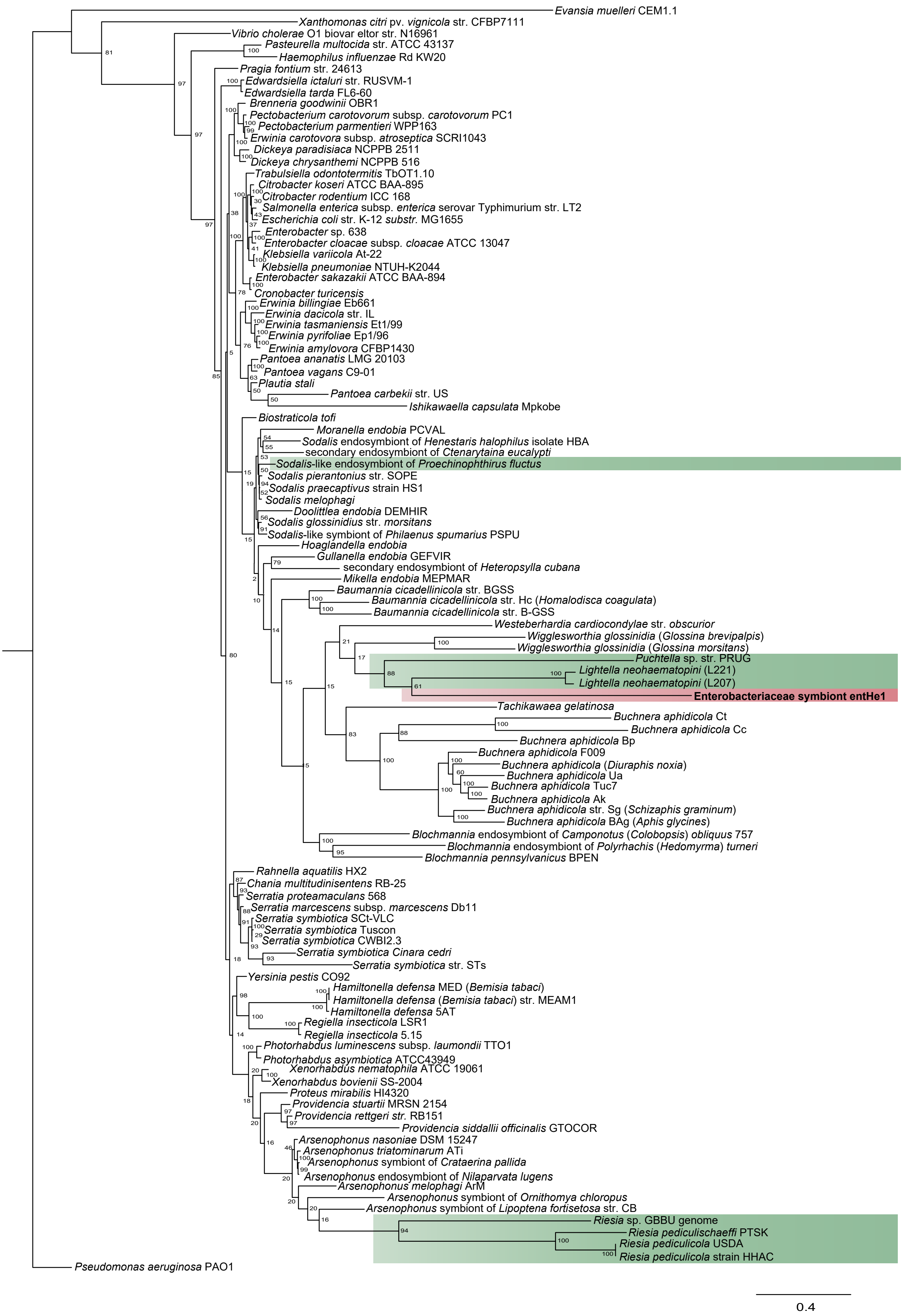

Supplementary figure S1: Phylogenetic tree inferred by Maximum likelihood (PhyML v.3.0) under the Q.plant +G+I+F evolutionary model. The numbers at nodes are bootstrap values obtained by 100 replications.
