# Supplementary material for: An obligate symbiont of Haematomyzus elephantis with a strongly reduced genome resembles symbiotic bacteria in sucking lice
Source: Appl Environ Microbiol. 2025 May 14;91(6):e00220-25. doi: 10.1128/aem.00220-25 (PMC12175528; doi:10.1128/aem.00220-25)
Supplement: Figure S2 — Bayesian inference tree. [file aem.00220-25-s0002.pdf]

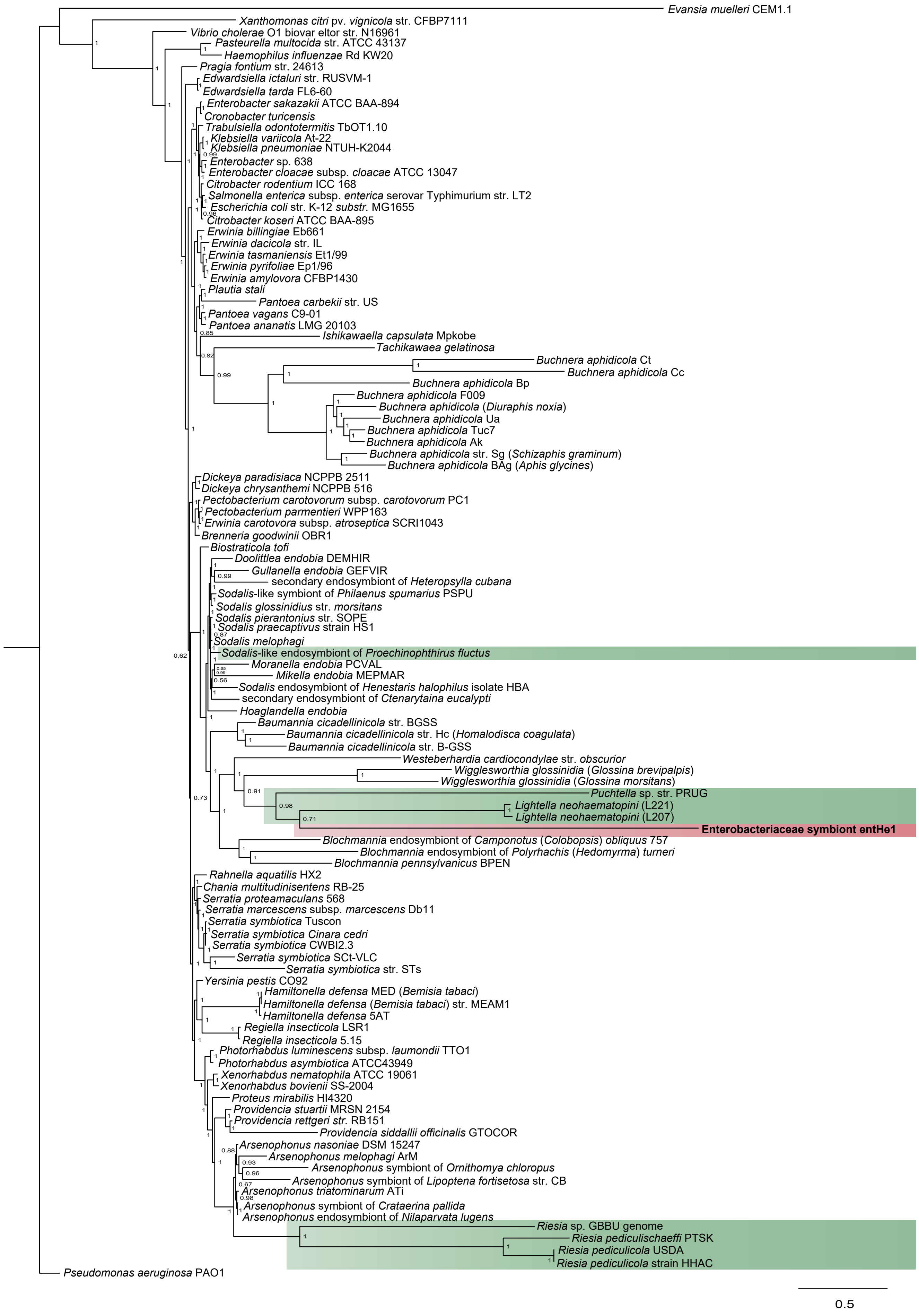

Supplementary figure S2: Phylogenetic tree inferred by Bayesian analysis (PhyloBayes MPI v.1.8) with CAT-GTR model. The numbers at nodes show posterior probabilities
